# Comparison of clinical and echocardiographic outcomes between mini-thoracotomy transatrial LuX-Valve transcatheter and surgical tricuspid valve replacement

**DOI:** 10.3389/fcvm.2024.1417757

**Published:** 2024-08-05

**Authors:** Lei Huang, Zhenxing Sun, Yu Cai, Yuji Xie, Ziming Zhang, Wei Sun, He Li, Lingyun Fang, Lin He, Li Zhang, Yali Yang, Jing Wang, Qing Lv, Yuman Li, Mingxing Xie

**Affiliations:** ^1^ Department of Ultrasound Medicine, Union Hospital, Tongji Medical College, Huazhong University of Science and Technology, Wuhan, China; ^2^ Clinical Research Center for Medical Imaging in Hubei Province, Wuhan, China; ^3^ Hubei Province Key Laboratory of Molecular Imaging, Wuhan, China

**Keywords:** transcatheter tricuspid valve replacement, surgical tricuspid valve replacement, tricuspid regurgitation, paravalvular leaks, tricuspid valve

## Abstract

**Background and aims:**

Transcatheter tricuspid valve replacement (TTVR) has recently emerged as a novel therapeutic approach for managing severe tricuspid regurgitation (TR). However, surgical tricuspid valve replacement (STVR) continues to be the predominant treatment modality. There are limited comparative data on both procedures. This study aimed to compare clinical and echocardiographic outcomes between patients who underwent mini-thoracotomy transatrial LuX-Valve TTVR and those who underwent STVR.

**Methods:**

This study prospectively collected patients with severe TR who underwent TTVR (*n* = 29) or isolated STVR (*n* = 59) at Wuhan Union Hospital from 2019 to 2022. All TTVR patients received the LuX-Valve via a mini-thoracotomy and transatrial approach. The clinical and echocardiographic outcomes were compared at 30-day and one-year follow-ups.

**Results:**

At baseline, patients with LuX-Valve TTVR had higher surgical risk scores and a greater proportion of right ventricular dysfunction compared with STVR. In the early postoperative period, the STVR group had a greater decrease in right ventricular function. Hospital length of stay (LOS), intensive care unit LOS, total procedure time, and tracheal intubation time were shorter in the TTVR than in the STVR group. The incidence of postoperative paravalvular leaks was higher among patients who underwent TTVR. Compared to the STVR group, the pacemaker implantation rate was lower in the TTVR group. During follow-up, the peak tricuspid valve velocity and mean gradient in the TTVR group were consistently lower than those in the STVR group. There was similar mortality between TTVR and STVR at 30-day and one-year follow-ups.

**Conclusions:**

The mini-thoracotomy transatria LuX-Valve TTVR has a higher incidence of paravalvular leaks and a lower rate of pacemaker implantation than STVR, with similar 30-day and one-year mortality rates. In some respects, mini-thoracotomy transatrial LuX-Valve TTVR may be a feasible and safe treatment option for specific populations, or it could potentially serve as an alternative therapy to supplement conventional STVR. Further follow-up is required to assess differences in long-term clinical outcomes and valve durability.

## Introduction

Clinically significant tricuspid regurgitation (TR) is common in the general population, with a prevalence of 0.55%, and its incidence increases with age, reaching a prevalence of 4% in people over 75 years of age (1). Moderate or greater TR is associated with increased mortality regardless of pulmonary artery systolic pressure, left ventricular ejection fraction, and right heart function (2, 3). Previous evidences demonstrated that untreated severe TR can decrease overall survival, with a 5-year survival rate of less than 50% (4–6). However, the treatment and management of this issue continue to be insufficient. Tricuspid valve surgery is recommended for treating severe TR based on the current guidelines (7, 8), but the timing for surgical intervention is still debated. About 86% of tricuspid valve surgeries are usually combined with left heart valve surgery, and isolated tricuspid valve surgeries are relatively rare (9). The mortality rate of isolated tricuspid valve surgery is high, due to the delayed referral, numerous complications, and the remodeling of the right ventricle (10–12).

Following the great success of transcatheter techniques in the aortic and mitral valve procedures, transcatheter tricuspid valve intervention (TTVI) has also recently developed rapidly as an option for symptomatic, inoperable, anatomically eligible patients with severe TR. A previous study showed no significant disparity in long-term survival rates between patients with isolated severe TR who underwent surgical intervention and those who solely received medical management (13). A clinical study utilizing propensity score matching showed that patients who received TTVI had lower rates of all-cause mortality and one-year rehospitalization than those who received medication (14). The 2021 ESC/EACTS Guidelines indicate that patients with severe TR who are not eligible for surgical intervention should consider transcatheter treatment at Heart Valve Centers with expertise in treating tricuspid valve disease (7). Previous studies have shown favorable results regarding the safety and efficacy of transcatheter tricuspid valve replacement (TTVR) (15–19). The ongoing advancement of TTVR technology offers an additional minimally invasive therapeutic option for individuals with severe TR. However, there are limited data comparing patients undergoing TTVR with those undergoing surgical tricuspid valve replacement (STVR) for severe TR.

Therefore, the present study aimed to assess the clinical and echocardiographic outcomes at 30 days and one year in a mini-thoracotomy transatria LuX-Valve TTVR cohort and compare these outcomes with those of conventional isolated STVR.

## Patients and methods

### Study population

Between January 2019 and December 2022, 88 patients underwent TTVR or isolated STVR at our institution. All the study subjects were divided into the TTVR group (*n* = 29) and the STVR group (*n* = 59).

All enrolled patients had severe, massive, or torrential TR. A multidisciplinary heart team selected each operation approach based on the comprehensive assessments of patient risk and anatomical findings. The inclusion criteria for the TTVR group were as follows: after a thorough discussion with the cardiac team, patients were deemed at high or extremely high risk for surgical procedures. Based on the comprehensive evaluations from echocardiographic and computed tomography analyses, the cardiac team determined that the patient's tricuspid valve anatomy was unsuitable for transcatheter edge-to-edge repair (TEER). This decision was informed by the presence of a coaptation gap exceeding 10 mm, significant leaflet tethering, or TR induced by a pacemaker. Conversely, the patient's tricuspid valve anatomy was deemed appropriate for LuX-Valve implantation. The exclusion criteria were as follows: patients with poor left or right ventricular function (left ventricular ejection fraction (LVEF) < 50%, tricuspid annular plane systolic excursion (TAPSE) < 10 mm or right ventricular fractional area change (RVFAC) < 20%), severe pulmonary hypertension (pulmonary artery systolic pressure [PASP] > 60 mm Hg [by right heart catheterization]), and untreated severe coronary artery disease.

The inclusion criteria for the STVR group were as follows: to minimize selection bias and obtain a clinically homogeneous cohort, the STVR group selected patients with isolated STVR. Patients with active infective endocarditis, those requiring concurrent surgery for coronary artery disease, those needing additional valve repair or replacement procedures, and those with combined congenital heart disease were excluded.

All the patients were transferred to the intensive care unit postoperatively and managed according to the approved postoperative care pathway. All the patients received anticoagulants or antiplatelet agents for a certain period after the procedure. The study was approved by the institutional ethics committee, and written informed consent was obtained from each participant.

### Echocardiographic evaluation

Echocardiographic evaluations were conducted at baseline, 30 days and one year after TTVR or STVR. Transthoracic echocardiography (TTE) and transesophageal echocardiography (TEE) were utilized for the screening phase, intraoperative guidance, and outcomes assessment. Echocardiographic parameters were obtained according to the definitional guidelines of the American Society of Echocardiography (20). Based on Simpson's method measurements of the apical 2-chamber and 4-chamber views, left ventricular (LV) volumes were determined, and the left ventricular ejection fraction (LVEF) was calculated. The biplane-modified Simpson's method was used to calculate left atrial (LA) volume. Single-plane disks were used to obtain right atrial (RA) volume. RV dimensions, RV end-systolic and RV end-diastolic areas, and RVFAC were measured in the RV-focused apical 4-chamber view. TAPSE was determined on M-mode recordings of the lateral tricuspid annulus in the RV-focused apical 4-chamber view. The PASP was calculated from the peak velocity of the TR jet by applying the Bernoulli equation and adding RA pressure according to the diameter and collapsibility of the inferior vena cava (21). RV-free wall longitudinal strain (RVFWLS) was assessed in the RV-focused apical 4-chamber view using Tom Tec Image-Arena (TOMTEC Corp., Chicago, IL, USA). The abnormal cutoff values for RV function parameters were set as TAPSE < 17 mm, RVFAC < 35%, and RVFWLS > −20% (20). RV dysfunction was defined as meeting any one of the above criteria.

The severity of TR was assessed using qualitative, semi-quantitative, and quantitative methods, as outlined in the American Society of Echocardiography guidelines (22). Furthermore, a 5-class grading scheme was employed for the grading of TR: mild, moderate, severe, massive, and torrential (23). Our study reported paravalvular leaks and total TR (including paravalvular leaks and transvalvular TR) incidences.

### Data collection and follow-up

The data were collected prospectively as a part of our institutional database. They included detailed information on patients' demographics, baseline clinical characteristics, laboratory and echocardiographic findings, intraoperative variables, and postoperative outcomes. The TRI-SCORE value was calculated based on the patient's preoperative characteristics, laboratory values, and echocardiographic parameters (24). The survival data for patients were obtained through telephonic communication with either the patients themselves or their respective family members.

### Operative techniques

All the transcatheter procedures were performed with the LuX-Valve TTVR system (Ningbo Jens Care Biotechnology Co., Ningbo, China), which were implanted through a minimally invasive thoracotomy and transatrial approach as previously described (15, 17) without the use of cardiopulmonary bypass (Supplementary Tables S1 and S2). Preprocedural multidetector CT and echocardiographic findings were utilized to ascertain valve size. All the procedures were conducted under general anesthesia and were facilitated by TEE and fluoroscopic guidance. TEE guided the catheter delivery, valve release, and valve position adjustment throughout the procedure.

The LuX-Valve System comprised a delivery system and a stent bioprosthesis. Specifically, the stent bioprosthesis was composed of four components: (1) a triple leaflet prosthetic valve with treated bovine pericardium; (2) a self-expanding nitinol valve stent coated with polytetrafluoroethylene fabric; (3) an interventricular septal anchoring component shaped like a bird's tongue; (4) two polytetrafluoroethylene-covered graspers. The stent bioprosthesis comed in 4 sizes (30–55 mm) and 8 skirt models for tricuspid annulus diameters between 25 and 50 mm. The diameter of the delivery system was 32 Fr, comprising three main components: the inner sheath, the outer sheath, and the core (15).

STVR procedures were conducted under extracorporeal circulation, utilizing median sternotomy and thoracotomy. The valve type was determined preoperatively, and the valve size was determined intraoperatively by calibration with a proprietary valve measuring device (Supplementary Table S1).

### Study endpoints

The primary endpoints of this analysis included all-cause mortality within 30 days and at one year, as well as readmissions due to heart failure (HF) within one year. Thirty-day mortality was defined as all-cause mortality within 30 days post-procedure, regardless of discharge. Secondary endpoints encompassed adverse events at 30 days and one year, including stroke, acute kidney injury with dialysis, bleeding requiring transfusion, reoperation for bleeding, permanent pacemaker implantation, myocardial infarction, deep sternal wound infection, device migration, TV reoperation, and valve thrombosis.

### Statistical analysis

Continuous variables are displayed as means ± SDs or medians (interquartile ranges) and were compared using a two-sample Student's *t*-test (for normally distributed data) or the Wilcoxon rank-sum test (for non-normally distributed data). Categorical variables are displayed as frequencies (percentages) and were compared using the chi-squared or Fisher exact tests. Paired *t*-tests were utilized to compare continuous variables before and after the operation within groups, while one-way analysis of variance was employed to compare continuous variables among the three groups. In the event of statistically significant overall results, *post hoc* multiple comparison tests were conducted using the Bonferroni correction. Kaplan-Meier analysis was used to analyze survival curves, and log-rank testing was used to compare them. Two-tailed tests were performed to test the hypotheses, and the significance level was set at 0.05. All statistical analyses were conducted using SPSS statistics version 26.0 (IBM, Armonk, New York).

## Results

### Baseline characteristics

Tricuspid valve replacement (TVR) was conducted in a total of 88 patients, with 29 patients receiving TTVR and 59 patients undergoing STVR. The baseline clinical characteristics are presented in Table 1. The TTVR patients were older and had exhibited a higher prevalence of prior left-sided valvular surgery, and higher TRI-SCORE and NT-pro BNP level than the STVR patients. Likewise, the TTVR group had a higher incidence of chronic atrial fibrillation, abdominal distension, and peripheral edema compared to the STVR group. Patients in the TTVR group exhibited more severe symptoms before the procedure, with a higher proportion in New York Heart Association (NYHA) functional class III/IV (79.3% vs. 52.5%, *P* = 0.011). The etiology of TR was primarily functional (75.9% in the TTVR group, 64.4% in the STVR group, *P* = 0.591). However, there were no significant differences in sex ratio, body mass index, incidence of hypertension or diabetes, prior pacemaker implantation, and hepatic and renal function parameters between the TTVR and STVR groups.

**Table 1:** Baseline characteristics.

Variables	TTVR (*n* = 29)	STVR (*n* = 59)	*P*-value
Female, *n* (%)	23 (79.3)	44 (74.6)	0.624
Age, years	67.7 ± 6.7	52 ± 10.4	<0.001
BMI, kg/m^2^	23.8 ± 5.5	22.5 ± 2.9	0.139
BSA, m^2^	1.6 ± 0.2	1.6 ± 0.1	0.895
Hypertension, *n* (%)	9 (31)	11 (18.6)	0.192
Diabetes, *n* (%)	7 (24.1)	5 (8.5)	0.055
Previous Left-sided valvular Surgery, *n* (%)	26 (89.7)	40 (67.8)	0.026
MVR	12 (41.4)	15 (25.4)	
AVR	1 (3.4)	1 (1.7)	
DVR	13 (44.8)	24 (40.7)	
Prior TV annuloplasty, *n* (%)	4 (13.8)	16 (27.1)	0.161
Prior pacemaker implantation, *n* (%)	1 (3.4)	3 (5.1)	>0.999
AF, *n* (%)	22 (75.9)	31 (52.5)	0.036
NYHA functional class, *n* (%)			0.011
II	6 (20.7)	28 (47.5)	
III	17 (58.6)	28 (47.5)	
IV	6 (20.7)	3 (5.0)	
TRI-SCORE, points	5.0 (4.0, 6.0)	3.0 (2.0, 5.0)	<0.001
Clinical symptoms			
Chest distress, *n* (%)	24 (82.8)	52 (88.1)	0.520
Abdominal distension, *n* (%)	14 (48.3)	14 (23.7)	0.020
Peripheral edema, *n* (%)	19 (65.5)	21 (35.6)	0.008
Biochemical parameters			
NT-pro BNP, pg/ml	358.0 (201.0, 639.0)	181.0 (104.0, 364.0)	0.002
Total bilirubin, U/L	16.1 (13.1, 23.1)	16.6 (12.6, 23.2)	0.828
Alanine aminotransferase, U/L	20.0 (15.0, 30.0)	19.0 (15.0, 29.5)	0.929
Aspartate aminotransferase, U/L	28.0 (24.0, 33.0)	28.0 (22.6, 36.5)	0.929
Alkaline phosphatase, U/L	75.0 (63.0, 92.0)	83.0 (58.0, 101.0)	0.558
Gamma-glutamyl transferase (U/L)	44.0 (29.0, 66.0)	54.0 (30, 93.5)	0.283
Lactate dehydrogenase, U/L	267.0 (221.0, 371.0)	267.0 (220.0, 326.0)	0.898
Creatine kinase, U/L	65.0 (46.0, 75.0)	63.0 (51.5, 79.5)	0.915
High-Sensitivity Troponin I	8.5 (4.4, 12.1)	6.5 (4.0, 9.8)	0.169
Glomerular filtration rate, ml/min/1.73 m^2^	94.4 (83.7, 101.4)	98.3 (88.6, 108.0)	0.134
**TR etiology**			0.591
Functional, *n* (%)	22 (75.9)	38 (64.4)	
Degenerative, *n* (%)	4 (13.8)	13 (22)	
Mixed, *n* (%)	3 (10.3)	8 (13.6)	

Values are number (%), mean ± SD for normally distributed numeric variables, or median (interquartile range) for non-normally distributed variables. BMI, body mass index; BSA, body surface area; MVR, mitral valve replacement; AVR, aortic valve replacement; DVR, double valves replacement; AF, atrial fibrillation; NT-pro BNP, N-terminal pro-brain natriuretic peptide.

### Procedural data and in-hospital outcomes

Table 2 summarises procedural characteristics and in-hospital outcomes. All the transcatheter procedures were performed with the LuX-Valve TTVR system, implanted via a minimally invasive right thoracotomy and transatrial approach. In the STVR group, 31 patients underwent thoracotomy, and 28 patients underwent median sternotomy. In the STVR group, 45 patients had a biological prosthesis implanted, while 14 patients received a mechanical prosthesis. Total procedure time was significantly shorter in the TTVR group compared to the STVR group [120.0 (100.0, 140.0) min vs. 240.0 (198.0, 261.0) min, *P* < 0.001].

**Table 2:** Procedural characteristics and in-hospital outcomes.

Variables	TTVR (*n* = 29)	STVR (*n* = 59)	*P-*value
Operative approach			
Transatrial, *n* (%)	29 (100)	–	–
Median sternotomy, *n* (%)	–	28 (47.5)	–
Thoracotomy, *n* (%)	–	31 (52.5)	–
Replacing valve type			
Biological prosthesis, *n* (%)	29 (100)	45 (76.3)	–
LuX-Valve	29	–	–
Medtronic	–	45	–
Mechanical prosthesis, *n* (%)	0	14 (23.7)	–
Sorin Carbomedics	–	2	–
St. Jude mechanical	–	5	–
Medtronic	–	7	–
Valve size, mm	29.1 ± 1.0	30.8 ± 1.8	0.007
Procedural outcomes			
CPB time, min		89.4 ± 35.0	–
ACC time, min (*n* = 14)		36.1 ± 14.0	
Total procedure time, min	120.0 (100.0, 140.0)	240.0 (198.0, 261.0)	<0.001
Technical success, *n* (%)	29 (100)	81 (100)	>0.999
Intraoperative mortality, *n* (%)	0	0	–
Conversion to sternotomy, *n* (%)	0	–	–
Paravalvular TR, *n* (%)	10 (34.5)	2 (3.4)	<0.001
^a^Residual TR ≥2+, *n* (%)	4 (13.8)	0	0.004
Periprocedural outcomes			
^#^In-hospital mortality, *n* (%)	1 (3.4)	5 (8.5)	0.380
Intensive care unit LOS, days	2.0 (1.0,3.0)	4.0 (3.0,5.0)	<0.001
Hospital LOS, days	13.0 (11.0, 15.0)	17.0 (13.0, 22.5)	0.007
Tracheal intubation time, days	1.0 (1.0, 1.0)	2.0 (1.0, 2.0)	<0.001
Postoperative 24-hour chest drainage, ml	200.0 (100.0, 300.0)	300.0 (200.0, 600.0)	0.001

Values are number (%), mean ± SD for normally distributed numeric variables, or median (interquartile range) for non-normally distributed variables; ACC, aortic cross-clamp; CPB, cardiopulmonary bypass; LOS, length of stay.

^a^ All were paravalvular leaks.

^#^ P-value was calculated by the Kaplan-Meier methods log-rank test.

There were no intraoperative deaths in either group. Intensive care unit LOS [2.0 (1.0, 3.0) days vs. 4.0 (3.0, 5.0) days, *P* < 0.001] and hospital LOS [13.0 (11.0, 15.0) days vs. 17.0 (13.0, 22.5) days, *P* = 0.007] were significantly shorter in the TTVR group. In addition, patients in the TTVR group had shorter tracheal intubation time and less postoperative 24-hour chest drainage than those in the STVR group. Immediate postoperative echocardiography showed residual TR ≥ 2 + in 4 patients, all of which were paravalvular leaks, and all occurred in the TTVR group. In-hospital mortality was similar between the TTVR and STVR groups (3.4% vs. 8.5%, *P* = 0.380).

### 30-day and one-year follow-up

Table 3 describes the 30-day and one-year mortality and adverse events for the TTVR and STVR groups. The STVR group had a higher frequency of 30-day adverse events than the TTVR group, but this difference did not attain statistical significance (23.7% vs. 13.8%, *P* = 0.533). During the 30-day follow-up period, 6 patients needed reoperation for bleeding, with 3 patients (10.1%) in the transcatheter group and 3 patients (5.1%) in the surgical group (*P* = 0.391). Although not statistically significant, it was noteworthy that 3 patients (5.1%) in the STVR group developed complete AV block and underwent permanent pacemaker implantation at the 30-day postoperative follow-up, which did not occur in the TTVR group. Furthermore, two patients experienced strokes, and one patient developed deep sternal wound infection in the STVR group, with no such events in the TTVR group.

**Table 3:** 30-Day and One-year follow-up.

Variables	TTVR (*n* = 29)	STVR (*n* = 59)	*P*-value
30-day follow-up			
^#^Mortality, *n* (%)	1 (3.4)	5 (8.5)	0.380
*No. of patients with adverse events, *n* (%)	4 (13.8)	14 (23.7)	0.533
Deep sternal wound infection, *n* (%)	0	1 (1.7)	>0.999
Reoperation for bleeding, *n* (%)	3 (10.3)	3 (5.1)	0.391
Bleeding requiring transfusion, *n* (%)	2 (6.9)	5 (8.5)	>0.999
Need for support device (ECMO, IABP, or others), *n* (%)	2 (6.9)	3 (5.1)	>0.999
Acute kidney failure with dialysis, *n* (%)	1 (3.4)	3 (5.1)	>0.999
Permanent pacemaker implantation, *n* (%)	0	3 (5.1)	0.548
Stroke, *n* (%)	0	2 (3.4)	>0.999
Myocardial infarction, *n* (%)	0	0	–
Valve thrombosis, *n* (%)	0	0	–
Device migration, *n* (%)	0	0	–
One-year follow-up			
^#^Mortality, *n* (%)	3 (10.3)	7 (11.9)	0.820
^#^HF readmission, *n* (%)	4 (13.8)	6 (10.2)	0.620
Permanent pacemaker implantation, *n* (%)	0	5 (8.6)	0.167
Stroke, *n* (%)	0	3 (5.1)	0.548
Myocardial infarction, *n* (%)	0	0	–
Valve thrombosis, *n* (%)	0	0	–
Device migration, *n* (%)	1 (3.4)	0	0.330

Values are number (%), mean ± SD for normally distributed numeric variables, or median (interquartile range) for non-normally distributed variables.

ECMO, extracorporeal membrane oxygenation; IABP, intra-aortic balloon pump.

^#^ P-value was calculated by the Kaplan-Meier methods log-rank test.

* n count is the number of patients with at least one adverse event.

Thirty days follow-ups inclusive of in-hospital events.

One-year follow-ups inclusive of in-hospital and 30-day events.

**Figure 1:**
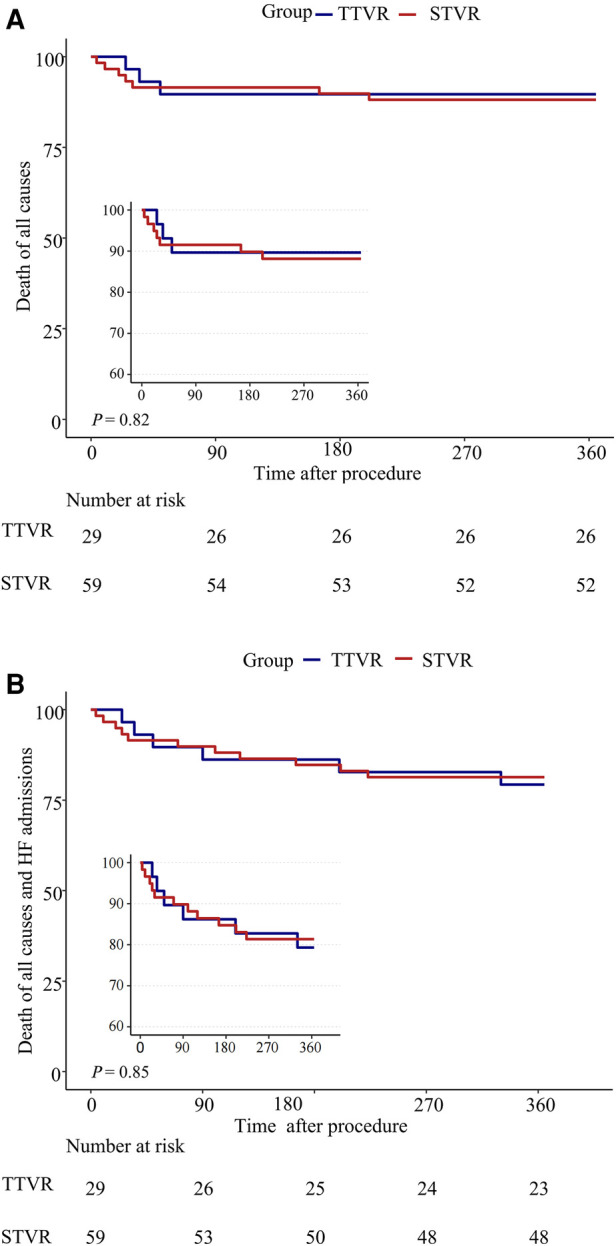
One-year outcomes of TTVR vs. STVR. (**A**) Death of all causes; (**B**) Death of all causes and HF readmissions.

One patient in the TTVR group was found to have device migration at 330 days postoperatively and subsequently underwent surgical tricuspid valve replacement. The incidence of readmission due to HF was comparable between the TTVR group and the STVR group at one-year follow-up, with 4 (13.8%) and 6 (10.2%), respectively. In addition, in the STVR group, 5 patients had permanent pacemakers implanted, and 3 patients had strokes, which did not occur in the TTVR group (TTVR 0% vs. STVR 8.6%, *P* = 0.167; TTVR 0% vs. STVR 5.1%, *P* = 0.548). Among the stroke patients, two underwent bioprosthetic valve replacement, and one underwent mechanical valve replacement. Mortality at 30 days (TTVR 3.4% vs. STVR 8.5%, *P* = 0.380) and one year (TTVR 10.3% vs. STVR 11.9%, *P* = 0.820) were similar between the TTVR group and the STVR group (Table 3, Figure 1).

### Echocardiographic characteristics

**Figure 2:**
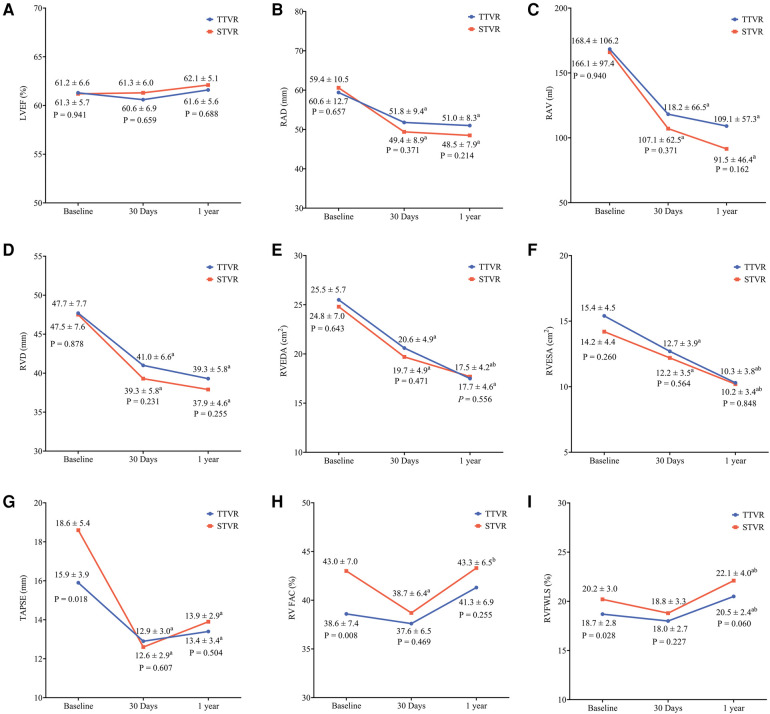
Changes in echocardiographic measures from baseline to 30 days and one year in the TTVR and STVR. (**A**) LVEF, left ventricular ejection fraction; (**B**) RAD, right atrial diameter; (**C**) RAV, right atrial volume; (**D**) RVD, right ventricular basal diameter; (**E**) RVEDA, right ventricular end-diastolic area; (**F**) RVESA, right ventricular end-systolic area; (**G**) TAPSE, tricuspid annular plane systolic excursion; (**H**) RVFAC, right ventricular fractional area change; (**I**) RVFWLS, RV free wall longitudinal strain. ^a^ indicates *P* < 0.05 comparison of baseline; ^b^ indicates *P* < 0.05 comparison of 30 days.

Echocardiographic data at baseline, 30 days, and one year following TTVR and STVR are shown in Table 4 and Figure 2. At baseline, the TTVR group had lower RVFAC, TAPSE and RVFWLS, a higher proportion of RV dysfunction (93.1% vs. 59.3%, *P* = 0.001), and higher PASP (44.8 ± 9.1 vs. 39.3 ± 10.5 mmHg, *P* = 0.019) as compared with the STVR group. At 30 days postoperatively, TAPSE decreased from baseline in both groups and did not significantly improve at one-year follow-up. Despite a decrease in RVFAC from baseline at 30 days, there was an improvement at the one-year follow-up. RVFWLS remained relatively stable in both groups at 30 days, with a significant improvement observed at one-year follow-up compared to the preoperative period. RV function was similar at 30 days and one-year follow-ups between the TTVR and STVR patients. Both groups demonstrated good right heart remodelling postoperatively. Additionally, patients who underwent TTVR and STVR had similar left and right heart sizes and LVEF at baseline, 30-day, and one-year follow-ups.

**Table 4:** Echocardiographic findings.

Variables	Baseline Echocardiogram			30-Day Echocardiogram			One-Year Echocardiogram		
	TTVR (*n* = 29)	STVR (*n* = 59)	*P*-value*	TTVR (*n* = 28)	STVR (*n* = 54)	*P* value*	TTVR (*n* = 26)	STVR (*n* = 52)	*P*-value*
Left heart									
LA anteroposterior diameter, mm	57.0 ± 18.8	52.0 ± 15.9	0.196	55.2 ± 13.6	49.1 ± 16.3	0.092	55.9 ± 11.6	50.2 ± 15.3	0.095
LA volume, ml	150.0 (86.3, 224.0)	103.0 (63.5, 184.7)	0.088	146.6 (90.1, 199.0)	106.0 (64.3, 172.8)	0.102	140.3 (83.9, 230.0)	106.5 (63.0, 163.3)	0.067
LV end-diastolic diameter, mm	46.1 ± 6.7	45.8 ± 6.1	0.877	47.2 ± 5.9	47.5 ± 4.5	0.797	46.8 ± 4.6	47.7 ± 6.0	0.512
LV end-diastolic volume, ml	96.4 ± 31.8	99.9 ± 27.5	0.696	98.6 ± 23.4	106.9 ± 24.6	0.146	100.2 ± 27.4	108.9 ± 25.7	0.168
LV end-systolic volume, ml	38.3 ± 12.5	40.4 ± 12.8	0.470	39.1 ± 11.9	41.3 ± 10.9	0.416	38.1 ± 10.5	41.4 ± 12.0	0.239
LVEF, %	61.3 ± 5.7	61.2 ± 6.6	0.941	60.6 ± 6.9	61.3 ± 6.0	0.659	61.6 ± 5.6	62.1 ± 5.1	0.688
Right heart									
RA diameter, mm	59.4 ± 10.5	60.6 ± 12.7	0.657	51.8 ± 9.4^a^	49.4 ± 8.9^a^	0.371	51.0 ± 8.3^a^	48.5 ± 7.9^a^	0.214
RV basal diameter, mm	47.7 ± 7.7	47.5 ± 7.6	0.878	41.0 ± 6.6^a^	39.3 ± 5.8^a^	0.231	39.3 ± 5.8^a^	37.9 ± 4.6^a^	0.255
RA volume, ml	138.0 (104.0, 201.9)	137.1 (108.0, 200.9)	0.940	100.5 (77.6,143.8)^a^	90.7 (66.9, 129.8)^a^	0.371	94.0 (71.2, 130.5)^a^	79.3 (61.8, 113.0)^a^	0.162
RV end-diastolic area, cm^2^	25.5 ± 5.7	24.8 ± 7.0	0.643	20.6 ± 4.9^a^	19.7 ± 4.9^a^	0.471	17.5 ± 4.2^a,^^b^	17.7 ± 4.6^a^	0.556
RV end-systolic area, cm^2^	15.4 ± 4.5	14.2 ± 4.4	0.260	12.7 ± 3.9^a^	12.2 ± 3.5^a^	0.564	10.3 ± 3.8^a,^^b^	10.2 ± 3.4^a,^^b^	0.848
RV FAC, %	38.6 ± 7.4	43.0 ± 7.0	0.008	37.6 ± 6.5	38.7 ± 6.4^a^	0.469	41.3 ± 6.9	43.3 ± 6.5^b^	0.206
TAPSE, mm	15.9 ± 3.9	18.6 ± 5.4	0.018	12.9 ± 3.0^a^	12.6 ± 2.9^a^	0.607	13.4 ± 3.4^a^	13.9 ± 2.9^a^	0.504
RVFWLS, %	−18.7 ± 2.8	−20.2 ± 3.0	0.028	−18.0 ± 2.7	−18.8 ± 3.3	0.227	−20.5 ± 2.4^a,^^b^	−22.1 ± 4.0^a,^^b^	0.060
IVC diameter, mm	22.7 ± 5.3	22.0 ± 6.8	0.650	20.4 ± 4.7	20.1 ± 3.6	0.722	18.4 ± 4.8^a^	18.3 ± 2.9^a^	0.864
Peak TV velocity, m/s^c^				1.2 (1.1, 1.5)	1.5 (1.3, 1.6)	0.024	1.3 (1.1, 1.5)	1.5 (1.4, 1.6)	0.014
Mean TV gradient, mm Hg^c^	–	–	–	3.0 (2.0, 4.0)	4.3 (3.2, 5.0)	0.002	3.0 (2.1, 4.3)	4.2 (3.2, 5.0)	0.012
Paravalvular TR, *n* (%)	–	–	–	9 (32.1)	2 (3.7)	<0.001	5 (19.2)	2 (3.8)	0.038
Tricuspid annular S-L, mm	41.0 ± 4.9	39.7 ± 5.4	0.188	–	–	–	–	–	–
PASP estimated, mm Hg	44.8 ± 9.1	39.3 ± 10.5	0.019	–	–	–	–	–	–
RV dysfunction, *n* (%)	27 (93.1)	35 (59.3)	0.001	–	–	–	–	–	–
Total TR severity			0.188			0.003			0.351
None/trace, *n* (%)	0	0		19 (67.9)	51 (94.4)		21 (80.8)	47 (90.4)	
Mild, *n* (%)	0	0		6 (21.4)	2 (3.7)		4 (15.4)	4 (7.7)	
Moderate, *n* (%)	0	0		3 (10.7)	1 (1.9)		0	1 (1.9)	
Severe, *n* (%)	11 (37.9)	33 (55.9)		0			1 (3.8)	0	
Massive, *n* (%)	8 (27.6)	15 (25.4)		0	0	0	0	0	
Torrential, *n* (%)	10 (34.5)	11 (18.6)		0	0	0	0	0	

Values are number (%), mean ± SD for normally distributed numeric variables, or median (interquartile range) for non-normally distributed variables. IVC, inferior vena cava; LA, left atrial; LV, left ventricular; LVEF, LV ejection fraction; RA, right atrial; RV, right ventricular; RV FAC, RV fractional area change; RVFWLS, RV free wall longitudinal strain; TAPSE, tricuspid annular plane systolic excursion; PASP, pulmonary artery systolic pressure.

Total TR including both paravalvular leaks and transvalvular TR.

^a^ indicates P < 0.05 comparison of baseline.

^b^ indicates P < 0.05 comparison of 30 days.

^c^ STVR data: bioprosthetic valve patients only.30-Day: n = 41; One-year: n = 39.

* P-value for TTVR group versus STVR group.

**Figure 3:**
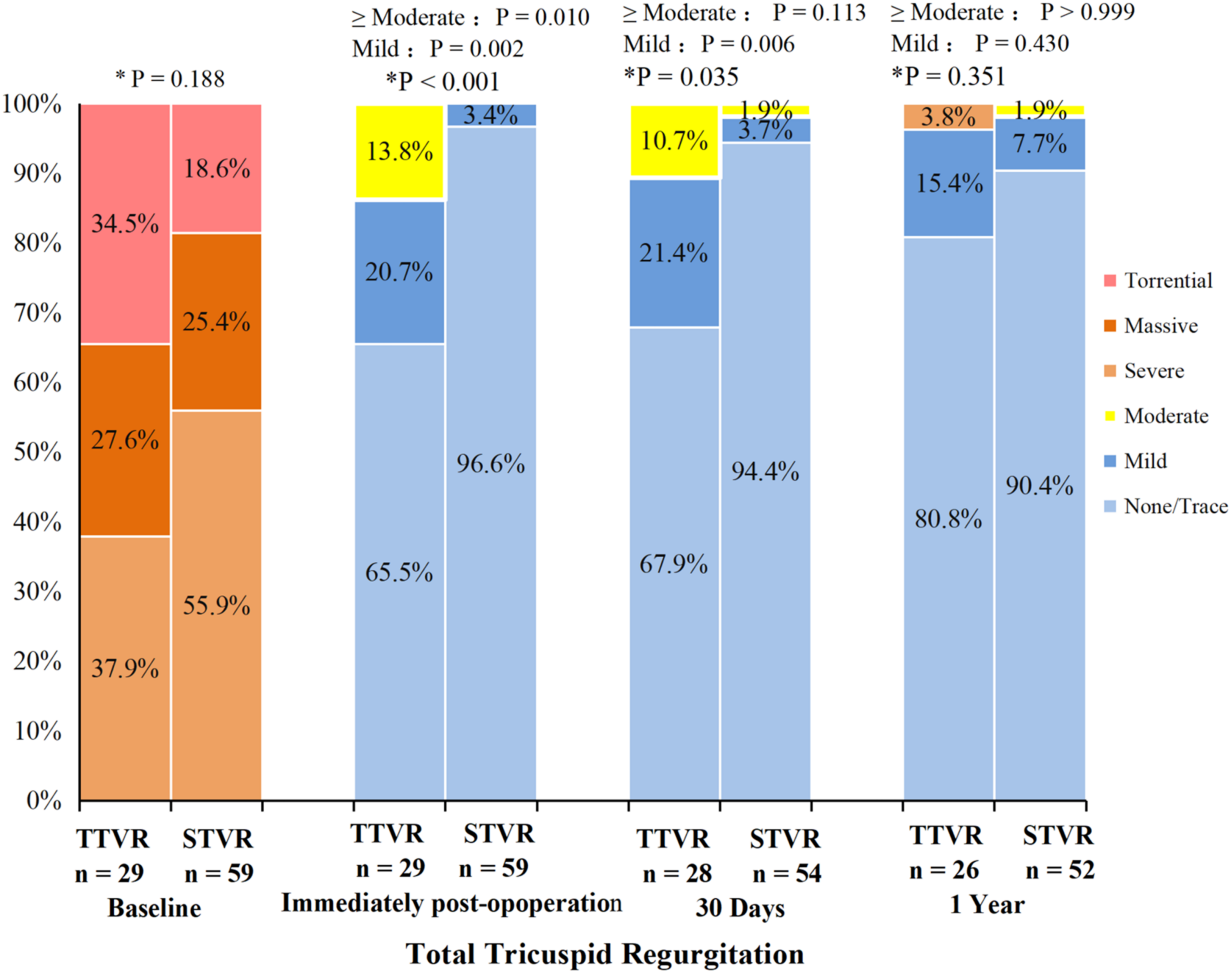
Total tricuspid regurgitation from baseline to 30 days and one year in the TTVR and STVR. * *P* value for TTVR group versus STVR group. Total TR including both paravalvular leaks and transvalvular TR.

With hemodynamics, the TTVR group showed lower peak tricuspid valve velocity and mean gradient than the STVR group at 30 days and one year. The peak tricuspid valve velocity and mean gradient remained stable in both groups over the 30-day and one-year follow-up periods. At baseline, the TR severity was similar in both TTVR and STVR groups, with all the patients having severe or greater TR. The distribution of total TR severity (paravalvular leaks and transvalvular TR) in the two groups at all time points is shown in Figure 3. Immediate postoperative TEE showed that residual TR ≥ 2 + and paravalvular leaks were more prevalent in the TTVR group than in the STVR group (13.8% vs. 0% and 34.5% vs. 3.4%, *P* < 0.05 for both). The percentage of ≥moderate paravalvular TR was higher after TTVR than STVR. However, both groups showed a noteworthy amelioration in the severity of TR immediately postoperatively compared to baseline. In the TTVR group, four patients had moderate paravalvular leaks, and three of them died during follow-up. One patient died within 30 days post-procedure from lung infection, and the other two died from right heart failure during the follow-up of 30 days to one year. The fourth patient with moderate paravalvular leaks also underwent STVR due to device migration within one year post-procedure. The TTVR group had a higher proportion of paravalvular leaks than the STVR group at both 30-day ansd one-year follow-ups (*P* < 0.05) (Table 4). At the one-year follow-up, the severity of total TR (paravalvular leaks and transvalvular TR) in the TTVR group did not appear to be significantly different from that in the STVR group (Table 4, Figure 3).

## Discussions

This study compared clinical and echocardiographic data between mini-thoracotomy transatrial LuX-Valve transcatheter and surgical tricuspid valve replacement and found the following major findings: (1) TTVR offers significantly shorter total procedure time, Intensive care unit LOS, tracheal intubation time, and hospital LOS compared to STVR; (2) compared with STVR, TTVR had a lower rate of pacemaker implantation, but a higher incidence of paravalvular TR; (3) despite higher preprocedural risk scores in the TTVR group, there was no significant difference in mortality between the TTVR and STVR groups at 30 days and one year.

Our results indicate that TTVR patients exhibited lower peak tricuspid valve velocity and mean tricuspid valve gradient when compared to STVR patients. It is probable that the differences in valve sizing and the slight ability for expansion of transcatheter valves, which is not feasible with a fixed-sized surgical sewing ring, are contributing factors. The long-term impacts of lower peak trans prosthetic velocity and mean trans prosthetic gradients on valve durability remain uncertain and will necessitate continued long-term follow-up.

The incidence and severity of paravalvular TR were higher after TTVR than after surgical intervention. The procedure outcomes showed four cases with residual TR ≥ 2+, all occurring in the TTVR group, and these were all paravalvular leaks. The TRILUMINATE trial indicated that patients with residual TR ≥ 2 + at 30 days had a threefold increased risk of experiencing death or hospitalization for HF (25). In our study, all three patients who died in the TTVR group had residual TR ≥ 2+, further demonstrating the association of this with mortality. At the one-year follow-up, the distribution of total TR (paravalvular leaks and transvalvular TR) was similar between the two groups. Moreover, the degree of TR in the TTVR group appeared to have improved compared to the 30-day follow-up. The observed improvement in the data could be attributed to deaths among patients with moderate paravalvular leaks in the TTVR group during the follow-up. Continued advancements in device technology and refined procedural strategies could potentially decrease the occurrence of paravalvular leaks.

The size of the left heart and LV function remained stable in both groups throughout the follow-up period. Both groups exhibited favorable right heart remodelling throughout the follow-up period, as evidenced by a noteworthy decrease in right heart dimensions and areas from the baseline. These findings align with prior research on transcatheter or surgical interventions (16, 26). Although the TTVR group had lower TAPSE and RV FAC at baseline compared to the STVR group, both groups manifested a reduction in these parameters after 30 days, with a more conspicuous decline in the STVR group. The baseline RV function of TTVR patients was worse due to chronic TR leading to RV myocardial injury and decreased RV function. This dysfunction became evident postoperatively following TR correction, as the preload decreased without initial compensation, resulting in a temporary decline (27). The decline observed in the surgical group may also be related to the use of cardiopulmonary bypass and pericardiotomy (28, 29). However, after one year, it seems to have improved with compensation. In patients with pre-existing severe RV dysfunction, the impact of TTVR on postoperative RV function may be less than that of STVR. The TTVR may be a favorable treatment option for patients with pre-existing severe RV dysfunction.

The present study reports a pacemaker implantation rate of approximately 8.4% (5/59) in the surgical group at the one-year follow-up, consistent with prior research (30). The EVOQUE System's one-year FIM Study revealed the rate of pacemaker implantation was 11% (3/27) (16) In a one-year FIM multicenter study of the LuX-Valve TTVR system, the rate of pacemaker implantation was 3.23% (1/31), and the implantation was considered device-independent (31). Notably, none of the patients in the transcatheter group in our study required postoperative pacemaker implantation, which could potentially be attributed to the unique design of the LuX-Valve. Previously reported implantation modalities for TTVR devices relied on radial forces between the device and the tricuspid annulus (32, 33). This type of fixation can lead to complications such as conduction block and right coronary artery impingement (34, 35). LuX-Valve utilizes a non-radial force design to reduce the incidence of these complications (36).

Despite the absence of significant differences between the two groups in bleeding requiring transfusion or reoperation for bleeding, bleeding remains a noteworthy adverse event that warrants attention. Our study indicated that two of three patients in the TTVR group who died required reoperation due to bleeding, which could be attributed to the transcatheter access approach. The transatrial approach may increase the likelihood of bleeding. Utilizing a large delivery sheath may require a transatrial approach, potentially leading to bleeding in the surgical or chest wall areas in individuals with coagulopathy. The presence of bleeding was a significant and independent prognostic indicator following the transcatheter intervention (37). As the approaches and planning of the TTVR procedure develop, it will become less invasive and more secure.

In our study, patients in the transcatheter group were significantly older and had a higher TRI-SCORE than those in the surgical group. However, it is essential to note that mortality from all causes at 30 days and one year was similar between the groups. Prior studies on TTVR using the GATE, EVOQUE, and LuX-Valve systems showed mortality ranging from 7% to 17.5% during the 6-month to one-year follow-up period (12–14, 29). The mortality for isolated STVR was between 10.9% and 12% and did not improve over time or with increasing surgical volume, which was consistent with the findings of our study (10, 11, 30). This finding provides additional evidence regarding the safety and effectiveness of TTVR. However, the potential applicability of TTVR to populations beyond high-risk groups and the possibility of expanding its indications necessitate further observation and clinical trials.

Currently, there have been only reports on the short-term efficacy of TTVR (16–18). However, there is still a lack of long-term data on the durability of prostheses in TTVR. Two-year observations of the EVOQUE system showed a 71% survival rate, accompanied by consistent symptom improvement and sustained efficacy in reducing TR (38). Furthermore, the LuX-Valve TTVR system demonstrated promising prospects for sustained efficacy and safety over a mid-to-long-term duration (39). However, these findings were based on a four-year follow-up involving a single patient. The data from our study suggest that TTVR may offer short-term efficacy equivalent to STVR, and may even be superior in some respects. Nevertheless, further longer-term follow-up studies are needed to assess the advantages and disadvantages of both approaches.

### Study limitations

Our study was a non-randomized, single-center study, which may introduce selection bias and limit generalizability. The small sample size also limits the power of our analyses. More reliable estimates in larger patient populations are needed. Several TTVR valves are available on the market, each utilizing different materials, designs, and implantation methods, and therefore, each carries various possible complications that may affect the treatment outcomes. Further technological advances (e.g., device iterations, smaller sheaths, transfemoral or transjugular approaches) may favorably influence the results of TTVR. The LuX-Valve transcatheter implantation via right atrial approach through minimally invasive thoracotomy carries a higher risk compared to the less invasive transfemoral/transjugular catheterization approach. It is noted that the LuX-valve Plus (transjugular approach), the next generation of LuX-Valve used in this trial, has entered clinical trials. Furthermore, prospective studies with larger cohorts are necessary to validate the long-term safety and efficacy of TTVR.

## Conclusions

Although the mini-thoracotomy transatrial LuX-Valve TTVR group has a higher surgical risk score than the STVR group, there is no significant difference in mortality between the two groups during the 30-day and one-year follow-ups. Compared to STVR, the LuX-Valve TTVR group has a higher incidence of paravalvular leaks and a lower pacemaker implantation rate. In some respects, the LuX-Valve TTVR may be a feasible and safe treatment option for specific population or it could potentially serve as an alternative therapy to supplement conventional STVR. However, long-term clinical outcomes and valve durability still require further follow-up studies.

## Data Availability

The raw data supporting the conclusions of this article will be made available by the authors, without undue reservation.
