# Supplementary material for: Comparison of clinical and echocardiographic outcomes between mini-thoracotomy transatrial LuX-Valve transcatheter and surgical tricuspid valve replacement
Source: Front Cardiovasc Med. 2024 Aug 5;11:1417757. doi: 10.3389/fcvm.2024.1417757 (PMC11330896; doi:10.3389/fcvm.2024.1417757)
Supplement: Supplementary file 1 [file Datasheet1.pdf]

Supplementary material

Supplementary Table S1 Prosthetic tricuspid valve types and sizes

	**TTVR group**		**n = 29**	**STVR group**		**n = 59**
**Valve model**	**LuX-Valve**	Size (mm)	29	**Biological prosthesis**	Size (mm)	45
	JS/TTVI-28-40	28	13		27	1
	JS/TTVI-28-50	28	16	**Medtronic**	29	11
	JS/TTVI-28-55	28	7		31	33
	JS/TTVI-30-50	30	9	**Mechanical prosthesis**		14
	JS/TTVI-30-55	30	7	Sorin Carbomedics	31	2
				St. Jude mechanical	29	2
					31	3
				Medtronic	29	2
					31	5

Supplementary Table S2 LuX-Valve Details

Model	Stent Diameter d (mm)	Support Section Length (mm)	Valve Height H (mm)	Total Length L(mm)	Annulus Size (CTA)
JS/TTVI-24-30	24	30	13	50	25-35
JS/TTVI-26-30	26	30	14	50	25-35
JS/TTVI-28-40	28	40	15	60	35-45
JS/TTVI-28-50	28	50	15	70	35-45
JS/TTVI-28-55	28	55	15	75	45-50
JS/TTVI-30-40	30	40	16	60	35-45
JS/TTVI-30-50	30	50	16	70	35-45
JS/TTVI-30-55	30	55	16	75	45-50

Supplementary Table S3 Echocardiographic findings in the TTVR group

Variables	Baseline	30-Day	One-year	P value	Baseline vs 30-Day	Baseline vs One-year	30-Day vs One-year
	(n = 29)	(n = 28)	(n = 26)		P value	P value	P value
**Left heart**							
LA anteroposterior diameter, mm	57.0 ± 18.8	55.2 ± 13.6	55.9 ± 11.6	0.093	0.669	0.799	0.870
LA volume, ml	150.0 (86.3, 224.0)	146.6 (90.1, 199.0)	140.3 (83.9, 230.0)	0.947	0.479	0.559	0.913
LV end-diastolic diameter, mm	46.1 ± 6.7	47.2 ± 5.9	46.8 ± 4.6	0.768	0.477	0.642	0.817
LV end-diastolic volume, ml	96.4 ± 31.8	98.6±23.4	100.2 ± 27.4	0.882	0.848	0.619	0.759
LV end-systolic volume, ml	38.3 ± 12.5	39.1 ± 11.9	38.1 ± 10.5	0.944	0.781	0.791	0.760
LVEF, %	61.3 ± 5.7	60.6 ± 6.9	61.6 ± 5.6	0.817	0.651	0.865	0.543
**Right heart**							
RA diameter, mm	59.4 ± 10.5	51.8 ± 9.4	51.0 ± 8.3	0.002	0.003	0.001	0.740
RV basal diameter, mm	47.7 ± 7.7	41.0 ± 6.6	39.3 ± 5.8	< 0.001	< 0.001	< 0.001	0.379
RA volume, ml	138.0(104.0,201.9)	100.5 (77.6,143.8)	94.0 (71.2, 130.5)	0.023	0.021	0.008	0.680
RV end-diastolic area, cm^2	25.5 ± 5.7	20.6 ± 4.9	17.5 ± 4.2	<0.001	< 0.001	< 0.001	0.028
RV end-systolic area, cm^2	15.4 ± 4.5	12.7 ± 3.9	10.3 ± 3.8	< 0.001	0.014	< 0.001	0.036
RV FAC, %	38.6 ± 7.4	37.6 ± 6.5	41.3 ± 6.9	0.141	0.608	0.151	0.056
TAPSE, mm	15.9 ± 3.9	12.9 ± 3.0	13.4 ± 3.4	0.004	0.002	0.010	0.630
RVFWLS, %	-18.7 ± 2.8	-18.0 ± 2.7	-20.5 ± 2.4	0.003	0.294	0.014	0.001
IVC diameter, mm	22.7 ± 5.3	20.4 ± 4.7	18.4 ± 4.8	0.009	0.094	0.002	0.142
Peak TV velocity, m/s	-	1.2(1.1,1.5)	1.3 (1.1,1.5)	-			0.903
Mean TV gradient, mm Hg	-	3.0 (2.0,4.0)	3.0 (2.1,4.3)	-			0.668
Paravalvular TR, n (%)	-	9(32.1)	5(19.2)	-			0.279
**Total TR severity**				< 0.001	< 0.001	< 0.001	0.218
None/Trace, n (%)	0	19 (67.9)	21 (80.8)				
Mild, n (%)	0	6 (21.4)	4 (15.4)				
Moderate, n (%)	0	3 (10.7)	0				
Severe, n (%)	11 (37.9)	0	1(3.8)				
Massive, n (%)	8 (27.6)	0	0				
Torrential, n (%)	10 (34.5)	0	0				

Values are number (%), mean ± SD for normally distributed numeric variables, or median (interquartile range) for non-normally distributed variables. IVC = inferior vena cava; LA = left atrial; LV = left ventricular; LVEF = LV ejection fraction; RA= right atrial; RV = right ventricular; RV FAC = RV fractional area change; RVFWLS = RV free wall longitudinal strain; TAPSE = tricuspid annular plane systolic excursion; PASP = pulmonary artery systolic pressure

Note: Total TR including both paravalvular leaks and transvalvular TR

Supplementary Table S4 Echocardiographic findings in the STVR group

Variables	Baseline (n = 59)	30-Day (n = 54)	One-year (n = 52)	P value	Baseline vs 30-Day P value	Baseline vs One-year P value	30-Day vs One-year P value
**Left heart**							
LA anteroposterior diameter, mm	52.0 ± 15.9	49.1 ± 16.3	50.2 ± 15.3	0.621	0.337	0.548	0.730
LA volume, ml	103.0 (63.5, 184.7)	106.0 (64.3, 172.8)	106.5 (63.0, 163.3)	0.846	0.575	0.478	0.880
LV end-diastolic diameter, mm	45.8 ± 6.1	47.5 ± 4.5	47.7±6.0	0.161	0.123	0.085	0.846
LV end-diastolic volume, ml	99.9 ± 27.5	106.9 ± 24.6	108.9±25.7	0.155	0.154	0.069	0.685
LV end-systolic volume, ml	40.4 ± 12.8	41.3 ± 10.9	41.4 ± 12.0	0.875	0.686	0.635	0.942
LVEF, %	61.2 ± 6.6	61.3 ± 6.0	62.1 ± 5.1	0.955			
**Right heart**							
RA diameter, mm	60.6 ± 12.7	49.4 ± 8.9	48.5 ± 7.9	< 0.001	< 0.001	< 0.001	0.661
RV basal diameter, mm	47.5 ± 7.6	39.3 ± 5.8	37.9 ± 4.6	< 0.001	< 0.001	< 0.001	0.255
RA volume, ml	137.1(108.0,200.9)	90.7 (66.9, 129.8)	79.3 (61.8, 113.0)	< 0.001	< 0.001	< 0.001	0.275
RV end-diastolic area, cm^2	24.8 ± 7.0	19.7±4.9	17.7 ± 4.6	< 0.001	< 0.001	< 0.001	0.066
RV end-systolic area, cm^2	14.2 ± 4.4	12.2 ± 3.5	10.2 ± 3.4	< 0.001	0.004	< 0.001	0.008
RV FAC, %	43.0 ± 7.0	38.7 ± 6.4	43.3 ± 6.5	< 0.001	0.001	0.803	0.001
TAPSE, mm	18.6 ± 5.4	12.6 ± 2.9	13.9 ± 2.9	< 0.001	< 0.001	< 0.001	0.094
RVFWLS, %	-20.2 ± 3.0	-18.8 ± 3.3	-22.1 ± 4.0	< 0.001	0.126	0.010	< 0.001
IVC diameter, mm	22.0 ± 6.8	20.1 ± 3.6	18.3 ± 2.9	0.009	0.114	0.003	0.647
Peak TV velocity, m/s[#]	-	1.5 (1.3,1.6)	1.5 (1.4,1.6)	-			0.785
Mean TV gradient, mm Hg[#]	-	4.3 (3.2,5.0)	4.2 (3.2,5.0)	-			0.772
Paravalvular TR, n (%)	-	2 (3.7)	2 (3.8)	-			> 0.999
**Total TR severity**				< 0.001	< 0.001	< 0.001	0.673
None/Trace, n (%)	0	51 (94.4)	47 (90.4)				
Mild, n (%)	0	2 (3.7)	4 (7.7)				
Moderate, n (%)	0	1 (1.9)	1 (1.9)				
Severe, n (%)	33 (55.9)		0				
Massive, n (%)	15 (25.4)	0	0				
Torrential, n (%)	11(18.6)	0	0				

Values are number (%), mean ± SD for normally distributed numeric variables, or median (interquartile range) for non-normally distributed variables. IVC = inferior vena cava; LA = left atrial; LV = left ventricular; LVEF = LV ejection fraction; RA= right atrial; RV = right ventricular; RV FAC = RV fractional area change; RVFWLS = RV free wall longitudinal strain; TAPSE = tricuspid annular plane systolic excursion; PASP = pulmonary artery systolic pressure

Note: Total TR including both paravalvular leaks and transvalvular TR

[#] bioprosthetic valve patients only.30-Day: n = 41; One-year: n = 39
